# Gut microbiota-derived SCFAs and MetS-related nephropathy

**DOI:** 10.3389/fnut.2025.1561271

**Published:** 2025-07-08

**Authors:** Xiaofang Tian, Li Sun, Shengjie Guo, Liying Yuan, Tang Zhang, Chengqian Huang, Tingting He, Qianfeng Jiang, Yizhou Zeng

**Affiliations:** ^1^Department of Nephrology, the First People's Hospital of Zunyi (The Third Affiliated Hospital of Zunyi Medical University), Zunyi, Guizhou, China; ^2^Guizhou Aerospace Hospital, Zunyi, Guizhou, China; ^3^Department of Cardiology, the First People's Hospital of Zunyi (The Third Affiliated Hospital of Zunyi Medical University), Zunyi, Guizhou, China; ^4^Department of Urology, the First People's Hospital of Zunyi (The Third Affiliated Hospital of Zunyi Medical University), Zunyi, Guizhou, China

**Keywords:** gut microbiota, short-chain fatty acids, metabolic syndrome, chronic kidney disease, energy metabolism

## Abstract

Metabolic syndrome (MetS) is a group of complex disorders characterized by abnormalities in the metabolism of proteins, fats, carbohydrates, and other substances in the human body. The kidney plays a vital role in these metabolic processes. Similarly, metabolic disorders can lead to renal damage, which can affect both its structure and function. The human intestinal tract possesses an abundant and diverse gut microbial community that significantly influences the physiology and pathology of the host. Growing evidence suggests that gut microbiota-derived metabolites exhibit multiple effects (anti-inflammatory, antioxidant, and improvement of lipid metabolism) in MetS. Particularly, considerable research has suggested that gut microbiota-derived short-chain fatty acids (SCFAs) have an intimate relationship with MetS-related nephropathy. The functions of SCFAs are involved in modulating energy metabolism, regulating immune and inflammatory responses, and inhibiting oxidative stress and mitochondrial damage, which are mainly through the activation of transmembrane G protein-coupled receptors (GPRs) and the inhibition of Histone deacetylase activity (HDAC). Regarding MetS-related nephropathy, therapeutic studies of SCFAs have been conducted in both clinical investigations and animal experiments. However, the role of SCFAs in kidney damage caused by various metabolic disorders has not been fully elucidated. The aim of this article is to review the role of SCFAs in MetS-related nephropathy, which will provide a prospective therapy strategy for MetS-related nephropathy.

## 1 Introduction

Metabolic syndrome (MetS) is a group of complex disorders involving abnormalities in the metabolism of the three major nutrients (proteins, fats, and carbohydrates) in the human body, including abdominal obesity or overweight, atherosclerotic dyslipidemia (hypertriglyceridemia and low HDL cholesterol), hypertension, and insulin resistance and/or glucose intolerance (1). Insulin resistance is the core of MetS and triggers a series of inflammatory responses, such as C-reactive protein and interleukin cytokines, causing damage to important organs (2). Similarly, the kidneys, as a crucial organ in metabolic processes, are sensitively impaired by metabolic disorders. Numerous previous studies have demonstrated that MetS augments an individual's susceptibility to developing chronic kidney disease (CKD) (3). Thus, MetS is considered a trigger for kidney injury in CKD, which magnifies the adverse impact of other insults.

Gut microbiota refers to the complex of various microorganisms in the human intestine. These microorganisms can produce a variety of metabolites, including short-chain fatty acids (SCFAs), ammonia, and sulfur compounds (4). The rich and diverse flora can use proteins, fats, carbohydrates, and other substances in the intestine to metabolize and release different metabolites. For example, protein metabolism can produce ammonia and sulfide, fat metabolism can produce fatty acids, and carbohydrate metabolism can produce sugars and SCFAs (5). Increasing data indicate that gut microbiota-derived metabolites exhibit multiple roles in the metabolic syndrome and may have significant deleterious and beneficial effects on host health. On the one hand, SCFAs are commonly acknowledged for their beneficial impacts on health. On the other hand, uremic toxins such as indoles, ammonia, and trimethylamine N-oxide, which are produced by the gut flora, are generally considered to be harmful substances (6). Specifically, extensive research has indicated that SCFAs generated from gut microbiota have a close association with nephropathy related to MetS.

The functions of SCFAs are involved in modulating energy metabolism, regulating immune and inflammatory responses, and inhibiting oxidative stress and mitochondrial damage, which are mainly achieved through the activation of transmembrane G protein-coupled receptors (GPRs) and the inhibition of histone acetylation (HDAC) (7). Currently, some therapeutic studies of SCFAs have been undertaken in both clinical investigation and animal experiments, such as those in obesity, diabetes, inflammatory bowel disease, hypertension, depression, and cancer (8–10). The growing number of therapeutic studies on SCFAs in clinical and fundamental research indicates a significant role for SCFAs in kidney diseases (11–13). Furthermore, emerging evidence indicates that SCFAs exerted some roles in MetS-related nephropathy (14–16). However, the underlying mechanism of SCFAs in MetS-related nephropathy has not been fully understood yet. This article aims to review the physiology and function of gut microbiota-derived SCFAs in MetS-related nephropathy and to propose prospective therapeutic strategies for MetS-related nephropathy.

## 2 MetS and kidney diseases

### 2.1 The definition of MetS

The concept of “MetS” was initially introduced by Grundy and colleagues in 2001 (17), which considerably arises from chronic inflammation caused by insulin resistance, along with disturbances in the metabolism of proteins, fats, carbohydrates, and other substances in the human body (18). Generally, the MetS is diagnosed based on five criteria proposed by the National Cholesterol Education Program-Adult Treatment Panel III (waist circumference, triglycerides, high-density lipoprotein, cholesterol, blood pressure, and glucose) (19). MetS is commonly influenced by internal genetic abnormalities or external surroundings. It is reported that a high-fat, high-carbohydrate diet structure, irregular lifestyle, and low physical activity are the main detrimental factors for MetS (20). There are various metabolic disorders in MetS, which mainly consist of insulin resistance, dyslipidemia, hypertension, obesity, high uric acid, and a high incidence of fatty liver (21). Following the increase in social burden and the spread of unhealthy living habits, the prevalence of MetS is gradually increasing, and MetS is regarded as an important contributor to a variety of diseases, including cardiovascular events, diabetes, cancer, and CKD (22–24).

### 2.2 The correlation between MetS and CKD

The kidney has a crucial role in metabolism, and an expanding body of evidence indicates that individuals with MetS are at a higher risk of developing CKD, which is characterized by impaired renal function and structural damage. Furthermore, the prevalence of MetS and its metabolic disorders is also much higher in patients with CKD than in the non-CKD population (25, 26). As shown in Table 1, there have been growing clinical investigations around the world evaluating the relationship between metabolic syndrome and CKD in the past decade. For example, a clinical study that enrolled 5800 individuals diagnosed with type-2 diabetes revealed that MetS served as an independent indicator for predicting the occurrence of CKD in new cases (27). A cross-sectional study conducted in 2012 found that the prevalence of CKD in China was 10.8% (10.2–11.3%), and the occurrence of renal damage was significantly associated with a variety of risk factors such as hypertension, diabetes mellitus, a history of cardiovascular disease, and hyperuricemia (28). Additionally, a survey of the 75,468 population in China also showed that the degree of MetS abnormality was positively correlated with the risk of CKD, and the prevalence of CKD in patients with MetS and those without MetS was 57% and 28%, respectively (29). Similarly, studies conducted in the United States have shown a correlation between MetS and a hastened advancement of CKD in individuals in stages 3 and 4 (30). In addition, a 10-year prospective cohort research was conducted on a sample of healthy individuals from the Republic of Korea to examine the connection between MetS and the occurrence of CKD (31). The findings revealed that MetS has emerged as an independent risk factor for the onset of CKD.

**Table 1:** The correlation between MetS and CKD.

**Years**	**Country**	**Type of study**	**Findings**	**References**
2008, 2012, 2014, 2022	China	Clinical study	—MetS served as an independent indicator for CKD, and the occurrence of renal damage was positively correlated with MetS-related risk factors.	(27–29, 35)
2013	United States	Clinical study	—MetS accelerates the progression of stages 3 and 4 CKD in patients.	(30)
2017	Republic of Korea	Clinical study	— MetS emerged as an independent risk factor for CKD incidence.	(31)
2018	Thailand	Clinical study	—The incidence of MetS in patients with CKD displays a higher incidence than that in patients without CKD.	(32)
2019	China	Clinical study	—Hypertension is not only the leading cause of CKD but also is positively associated with the prevalence of CKD.	(33)
2020	China	Clinical study	—overweight/obesity significantly increased the risk of CKD.	(34)
2023	Iran	Clinical study	—There is no obvious relationship between abdominal obesity and reduced HDL and the incidence of CKD.	(36)
2018, 2021	United States, Austria, Germany	Clinical study	—The higher “MetS” score and the susceptibility and incidence of developing CKD.	(38, 39)
2022	China	Clinical study	— The components of MetS were significantly correlated with CKD.	(40, 41)
2021	Italy	Clinical study	—MetS and not obesity is associated with kidney damage.	(42)
2023	United States	Animal study	—MetS has increased susceptibility to CKD and has a higher risk of mortality	(47)
2017	United States	Animal study	—MetS induced apparent renal inflammation and fibrosis.	(48)
2020	Spain	Animal study	—MetS induced pathological changes in the kidney.	(49)

MetS, metabolic syndrome; CKD, chronic kidney disease.

Recently, there have been ongoing updates in domestic research on MetS and CKD aimed at providing more evidence in this field. Research in Thailand reported that the incidence of MetS in patients with CKD was 71.3%, displaying an evident higher incidence than that in patients without CKD (32). A multi-center and cross-sectional study involving 2,484 patients found that hypertension is not only the leading cause of CKD but also is positively associated with the prevalence of CKD (33). A cohort study of 15,229 participants from 2008 to 2013 demonstrated a significant increase in the risk of CKD due to overweight/obesity (34), and also found that MetS was independently associated with renal dysfunction (35). Interestingly, an investigation that recruited 8,987 participants showed that MetS promotes the development of CKD and is correlated with some strong indicators, including hypertension, diabetes, and age. There is no obvious relationship between abdominal obesity and reduced HDL and the incidence of CKD (36). In recent years, the “MetS score” and the “MetS factor” have been applied to identify MetS and its components (37). Previous studies confirmed that the higher the “MetS score” obtained, the higher the susceptibility and incidence of developing CKD (38, 39). When the components of MetS were analyzed separately with the incidence of CKD, several clinical investigations suggested that some metabolic components were still significantly correlated with CKD, including age, body mass index, waist circumference, systolic and diastolic blood pressure, serum triglyceride, serum glucose, serum uric acid, and C-reactive protein. Therefore, targeted screening and intervention tests among individuals were feasible and highly necessary (40–42). Considering the close correlation between MetS and CKD, gaining more understanding of their relationship can help us to further investigate the underlying mechanism.

### 2.3 The pathogenesis of MetS-related nephropathy

Insulin resistance is the critical feature of MetS, and insulin receptors are expressed in podocytes, tethered cells, renal microvascular endothelial cells, and tubular epithelial cells of the kidney (43). It is indicated that renal tissues in the MetS population predominantly exhibit chronic lesions, including varying degrees of glomerulosclerosis, tubular atrophy, interstitial fibrosis, and atherosclerosis (44, 45). It is suggested that glomerulosclerosis, cystic wall thickening, increased glomerular volume, and atherosclerotic vitriform degeneration of small renal arterioles were more pronounced in patients with MetS. In addition, the incidence of IgA nephropathy and focal segmental glomerulosclerosis was significantly higher in patients with MetS, and obesity may be an independent risk factor for IgA nephropathy (46). Additionally, multiple animal experiments have explored the changes and potential mechanisms of MetS-related nephropathy. For example, animal research confirmed that MetS increased susceptibility to CKD and risk of mortality (47). Moreover, significant renal inflammatory and fibrotic changes were observed in a porcine model of MetS (48). Similarly, it is reported that mesangial expansion, nodular glomerulosclerosis, and glomerulomegaly were validated in a model of metabolic syndrome that used Iberian pigs fed with fat-enriched food (49). The pathogenesis of MetS-related renal damage is very complex; obesity, insulin resistance, dyslipidemia, and hypertension usually serve as inducers to promote the progress of CKD. However, the underlying mechanism of MetS-related nephropathy has not been fully understood yet. It has been suggested that the activation of RAAS, production of inflammatory factors, and induction of reactive oxygen species (ROS) are crucial in the development of MetS (50, 51). Transforming growth factor-β_1_ (TGF-β_1_) is increased in renal inflammation and renal fibrosis, which contributes to the progression of MetS-related nephropathy (52, 53). TGF-β_1_ could activate the tumor suppressor p53 in diabetic insults; targeting p53 may have the efficacy for protecting MetS-related nephropathy (54). Similarly, several studies have suggested that the sterol regulatory element binding protein-1 (SREBP-1) is participating in promoting renal fibrosis of diabetic nephropathy (55). SREBP inhibition does not have a significant effect on ameliorating renal damage in the early stage of diabetic nephropathy (56).

Furthermore, lots of other potential molecular mechanisms were still explored in various studies recently, including insulin-like growth factor-1 (IGF-1), connective tissue growth factor (CTGF), HIF-1α, AMPK, and Nrf2 pathways, which were found to be correlated with MetS-related nephropathy (57–60). *In vivo* and *in vitro* experiments revealed that activation of the NLRP3 inflammasome exacerbated metabolic kidney injury by regulating ROS and mitochondrial damage (61, 62). Interestingly, emerging evidence suggested that gut microbiota and its metabolites played some roles in MetS-related kidney diseases (63–65). For example, an animal investigation on hyperuricemia-related nephropathy was performed and found that gut microbiota exhibited protective roles against renal inflammation in mice (66). Of course, there are numerous studies about the relevant mechanisms of metabolic kidney injury around the world, and we just depict the tip of the iceberg. A better understanding of the mechanisms of MetS-associated renal damage could guide potential therapeutic targets to prevent the development of MetS-related nephropathy.

## 3 Gut microbiota-derived SCFAs

### 3.1 The overview of intestinal flora

The intestinal microbiota has a large number of 10 trillion or even 100 trillion microorganisms, including bacteria, fungi, and viruses (67, 68), and the development of high-throughput sequencing technology has revealed that the gut flora contains far more genes than the human genome, leading to the term “second human genome” (69). In healthy adults, the phyla *Firmicutes* and *Bacteroidetes* are predominant. Factors such as age, medications, and allergens can affect the structure and number of intestinal flora, as well as cause changes in metabolites (70). Diseases such as obesity, type 2 diabetes, atherosclerosis, inflammatory bowel disease, and cancer have been linked to disruptions in the gut flora (71, 72). The term “gut-kidney axis” was originally used in 2011 to describe a theory that postulates the existence of a crosstalk-like relationship between the kidneys and the intestines that is controlled in both directions to create a gut–kidney axis balance and to maintain health (73). In recent years, studies have suggested a close relationship between the gut microbiota and kidney diseases. For example, during the course of CKD, uremic toxins gradually accumulate, which may be accompanied by chronic intestinal inflammation and epithelial dysfunction, promoting the translocation and structural changes of the intestinal flora. The change in the gut microbiota will, in turn, exacerbate the increase of uremic toxins, resulting in a vicious cycle within the gut–kidney axis (74). Some studies collected relevant literature from PubMed over the past 10 years for analysis and found that the impact of metabolomics in chronic kidney disease is higher than that of proteomics and transcriptomics. Moreover, the research results observed the enrichment of *Eggerthella lenta, Enterobacteriaceae*, and *Clostridium spp*., as well as the depletion of *Bacteroides eggerthii, Roseburia faecis*, and *Prevotella spp*. in the CKD model (75). Some studies analyzed the role of gut microbiota in acute kidney injury (AKI) (76) and also summarized the possible pathogenic pathways and mechanisms involved in the transition from AKI to CKD due to gut microbiota dysregulation (77). In addition, some literature reports that the gut microbiome may play a role in maintaining oxalate homeostasis and nephrolithiasis. Gut microbiota may also interact with symbiotic bacterial species in the urinary microbiome to jointly affect crystal formation and induce stone growth in the kidney (78). Furthermore, recent studies have particularly emphasized the close association between IgA nephropathy and gut microbiome dysregulation. Gut microbiome dysregulation is associated with an abnormal gut mucosal immune system, leading to the production of abnormal IgA1 antibodies by the body, which further form immune complexes and deposit in the kidneys, triggering kidney inflammation and damage (79). As a huge biological community, the gut microbiota plays a non-negligible role in kidney diseases, but the specific mechanism is not yet clear, which is worthy of our continuous in-depth exploration and discussion.

### 3.2 The definition of SCFAs

The intestinal flora produces a variety of metabolites through the breakdown of nutrients, including indolephenol sulfate, p-cresol sulfate, trimethylamine oxide, horse uric acid, and SCFAs. SCFAs are the products of fermentation of dietary fibers by the intestinal flora at the site of the cecal colon, which primarily consists of acetate, propanoate, butyrate, isobutyrate, valerate, isovalerate, hexanoate, and isohexanoate (7). SCFAs are usually present in the gut and can be absorbed into the bloodstream (80). Finally, the fraction of SCFAs was excreted via the breath, urine, and feces (81, 82). After entering the circulatory system, SCFAs could be distributed in many peripheral tissues, such as the heart, kidney, skin, and sympathetic ganglia (83–86), and modulate biological processes through binding to G-protein coupled receptors (GPR41, GPR43, and GPR109A) or inhibiting histone acetylation (87). GPR41, GPR43, and GPR109 are expressed in a variety of cells and tissues, serving as vital receptors activated by SCFAs for participating in some protective effects (88–90). Dietary fiber modulates microbial composition and influences the production of SCFAs (91), and alterations in gut flora directly affect acetate, propionate, and butyrate levels. The role of SCFAs in diseases remains controversial, and the underlying mechanisms involved are still unclear. For example, butyrate is thought to have antitumor, antifibrotic, and anti-inflammatory activities (92, 93). However, some studies do not support their protective role in disease (94).

### 3.3 The role of SCFAs in MetS

The core of MetS development is insulin resistance, which could be affected by gut microbiota-derived metabolites. To a certain extent, SCFAs play an important role in MetS. Similar to the diverse effects of SCFAs, considerable studies on SCFAs have been carried out in both clinical and animal studies. Although there may be a few discrepant opinions about the role of SCFAs in some diseases, the current mainstream view tends to consider SCFAs to play a beneficial role for individuals. Lucas S *et al*. reported that treatment of mice with SCFAs or feeding with a high-fiber diet increases bone mass and prevents postmenopausal and inflammation-induced bone loss (95). Moreover, accumulating evidence has suggested that SCFAs exhibit pivotal effects in MetS. Clinical research has found that the changes in SCFAs in the body after conservative weight loss and surgical intervention in obese patients are very interesting. It shows that the total and relative amounts of acetate, propanote, and butyrate decrease while the total and relative amounts of isobutyrate, isovalerate, and isohexanoate increase. This result suggests a shift in the proteolytic fermentation pattern, which has an adverse effect on health. SCFAs are related to diet, and the adverse effects can be offset through dietary intervention (96). SCFAs utilized in animal models of obesity have been shown to reduce lipogenesis and inhibit body weight gain, potentially by enhancing triglyceride hydrolysis and FFA oxidation in adipose tissue (97, 98). In addition, the levels of SCFAs have a close correlation with blood pressure (99). Recently, research showed that fecal levels of SCFAs were significantly decreased in patients with preeclampsia; pregnant rats of hypertension were utilized to explore the further mechanism, which demonstrated that SCFAs could directly regulate blood pressure and improve hypertension (100). The central features of diabetes are chronic inflammation and peripheral insulin resistance; accumulating evidence indicated that SCFAs could improve insulin sensitivity and prevent inflammation in *in vivo* and *in vitro* models of diabetes (101). However, the changes in SCFAs in preeclamptic patients are inconsistent with those in obese and other metabolic syndrome patients, which is a point worthy of great attention. We speculate that the shift in the metabolic pattern of nutrients may prompt changes in the gut microbiota structure, leading to a reduction in SCFA-producing bacteria. This may then give feedback and regulate the body to produce more SCFAs, resulting in an increase in the level of SCFAs in feces. At the same time, relatively high content of SCFAs in feces may also be secondary to a reduction in the intestinal absorption of SCFAs, leading to an obstacle in the process of SCFAs being absorbed into the bloodstream (102, 103). In preeclamptic patients, there is a relative disorder in the gut microbiota, such as a decrease in the abundance of *Firmicutes* and an increase in the abundance of *Proteobacteria*. Moreover, the fecal SCFA level in preeclamptic patients is positively correlated with the abundance of *Firmicutes*. The increase in the abundance of Proteobacteria may promote an increase in the production of LPS, thus triggering an excessive inflammatory response, resulting in a decrease in the relevant SCFAs in feces (104). However, the mechanisms involved in these differential manifestations are relatively complex and are not clear at present, awaiting further research.

### 3.4 The role of SCFAs in kidney diseases

**Figure 1:**
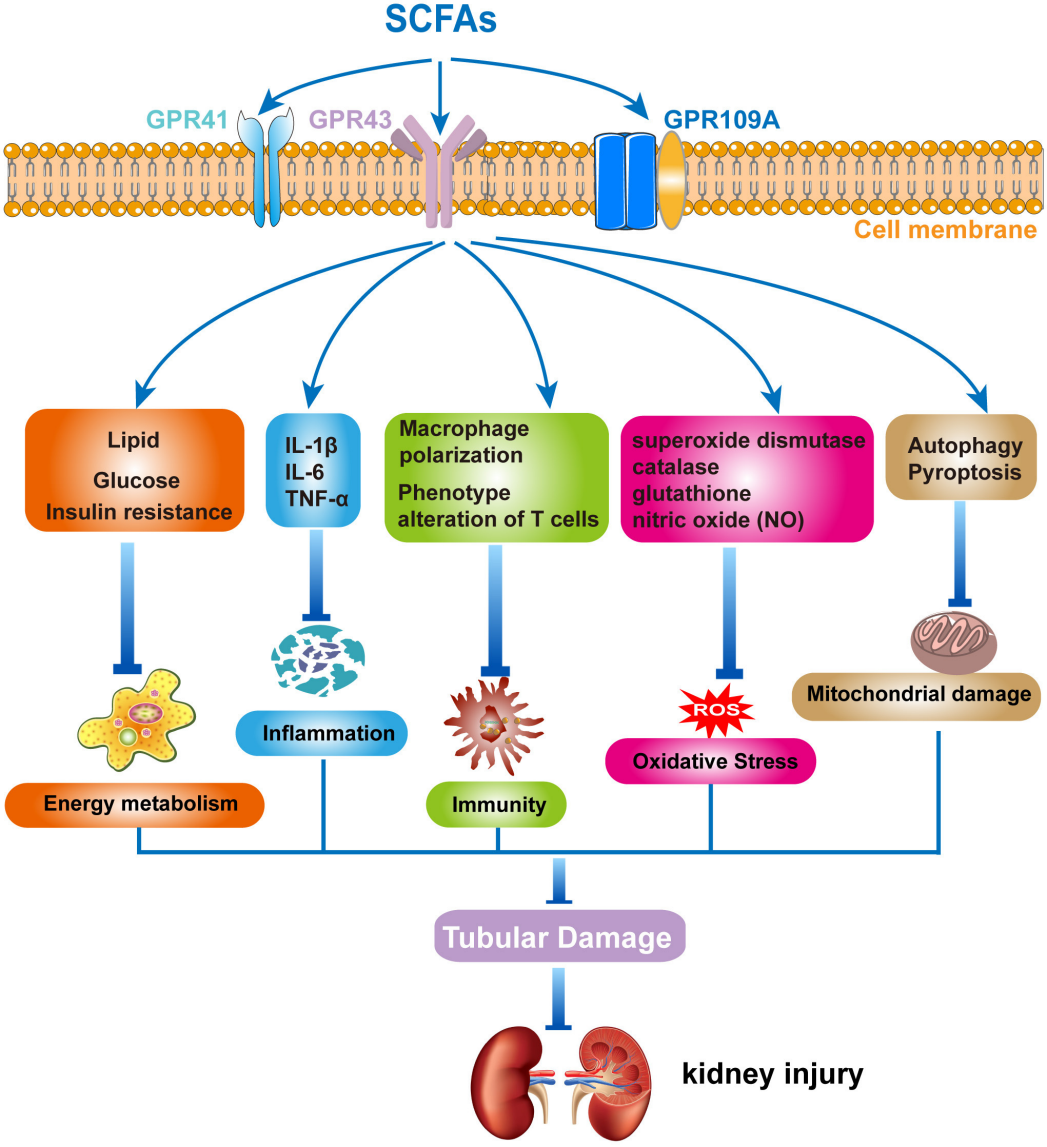
The mechanism of short-chain fatty acids (SCFAs) in kidney diseases. Gut microbiota-derived SCFAs exert protective effects in kidney diseases by modulating energy metabolism, regulating inflammation and immune response, and inhibiting oxidative stress and mitochondrial damage.

Recently, accumulating evidence has suggested that SCFAs have an imitated relationship with CKD. Metagenomic analysis of the gut microbiome demonstrated that the evident changes of SCFAs can be observed in early CKD (105). Growing animal experiments illustrated that SCFAs exerted a substantial role in preventing the progress of CKD (106–111). However, the underlying mechanisms of SCFAs in renal disease remain unclear. It is currently proposed that SCFAs regulate the development of renal diseases primarily through the following aspects: (1) the effect of SCFAs on energy metabolism, where SCFAs play an important regulatory role in energy metabolism, including lipid, glucose, and insulin (112, 113). The metabolism of lipids, glucose, and insulin is regulated by SCFAs via activating GPRs and inhibiting HDACs (114, 115). These regulatory actions could have a significant impact on the onset and progression of CKD; (2) the effect of SCFAs on immunity and inflammation: SCFAs have anti-inflammatory effects and affect the immuno-inflammatory process (116). A clinical investigation in children with CKD found that gut barrier dysfunction and microbial metabolite imbalance apparently mediated the production of pro-inflammatory factors (IL-1β, IL-6, and TNF-α), as well as altered T-cell phenotype (117). Moreover, SCFAs significantly increased the level of M2 macrophage polarizing factor, limiting the progression of renal fibrosis (118). Thus, SCFAs may affect the development of renal diseases by modulating immuno-inflammatory processes; (3) the effect of SCFAs on oxidative stress and mitochondrial damage: superoxide dismutase, catalase, glutathione, and nitric oxide (NO) all contribute to ROS production in the process of oxidative stress. Treatment with SCFAs ameliorated the proximal tubule injury, which was associated with oxidative stress (84). In addition, autophagy and pyroptosis are critical for the accumulation of mitochondrial damage involved (119, 120). Previous studies revealed that SCFAs played a crucial role in the prevention of CKD, including the promotion of mitochondrial biogenesis and the reduction of mitochondrial damage (121, 122). Therefore, gut microbiota-derived SCFAs play some roles in kidney diseases, including energy metabolism, immune inflammation, oxidative stress, and mitochondrial damage, affecting the occurrence and development of kidney diseases (Figure 1). However, there are many issues that still require further investigation, such as the specific signaling pathways, as well as the type and distribution of receptors, and how SCFAs regulate immune-inflammatory responses and interact with other cytokines. Based on the above-mentioned roles of SCFAs in MetS and kidney diseases, we speculate that SCFAs play an important role in MetS-related nephropathy. Then, we will comprehensively analyze the progress of SCFAs in MetS-related nephropathy.

## 4 SCFAs in MetS-related nephropathy

**Figure 2:**
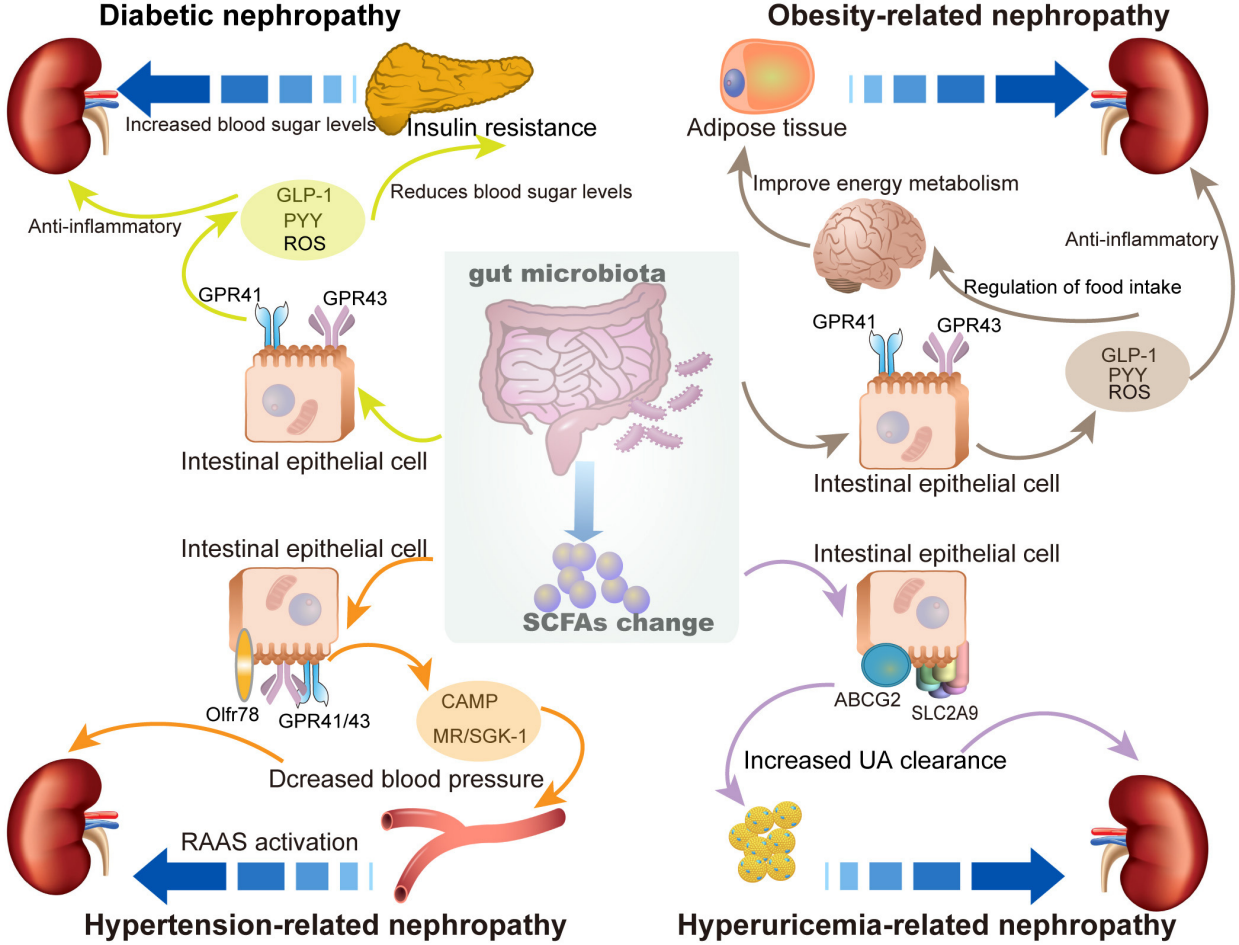
The role of SCFAs in metabolic syndrome (MetS)-related nephropathy. The enrichment of SCFAs exhibits multiple protective effects to prevent SCFAs MetS-related nephropathy, including diabetic nephropathy, obesity-related nephropathy, hypertension-related nephropathy, and hyperuricemia-related nephropathy.

Abundant SCFA production can regulate the health status of the host and exert multiple protective effects against a variety of diseases, including diabetes, obesity, cardiovascular disease, and kidney diseases. This beneficial effect is due to the contribution of SCFAs in modulating energy metabolism, regulating inflammation and immune response, and inhibiting oxidative stress and mitochondrial damage. Studies on the role and related mechanisms of SCFAs in the prevention and treatment of MetS-related nephropathy are summarized in Figure 2.

### 4.1 Diabetic nephropathy

Diabetes mellitus (DM) is a group of metabolic diseases evaluated by hyperglycemia and insulin resistance, which often causes various chronic microvascular damage. Diabetic nephropathy (DN) is known as a serious microvascular complication of diabetes and is the leading cause of end-stage renal disease (ESRD) (123, 124), which is characterized by glomerulosclerosis, tubular atrophy, and fibrosis, concomitant with oxidative stress and NF-κB signaling activation (125). The pathological process of DN is extremely complex; particularly, the aberrant immune system and chronic inflammation have been regarded as pivotal regulators in the pathophysiological process and promote the onset and progression of DN (126, 127). Dysbiosis of gut microbiota is present in patients and rodents with DN, especially the decreased SCFAs-producing bacteria. The abundance of the gut microbiota in DN patients is reduced, and the relative abundance of *Firmicutes* is lower than that in the general healthy population (128). A 2022 study conducted a meta-analysis of the gut microbiota composition in healthy individuals and those with DN, DM, and non-diabetic nephropathy and found that the average abundance of *Firmicutes* in the gut microbiota of DN patients was reduced, while the average abundance of *Actinobacteria* increased. In addition, compared with patients without DN, the abundance of specific genera (such as *Hungatella, Bilophila*, and *Escherichia*) in the gut microbiota of DN patients changed significantly (129). Over the last few years, there has been an increasing amount of study focusing on the role of SCFAs in the DN (130). A clinical investigation provided evidence that the concentration of serum and fecal SCFA levels (fecal levels in particular) were lowered in individuals with DN, which are negatively correlated with renal function (131). Moreover, 308 subjects with type 1 diabetes maintained normal renal function, whose subsequent detection of metabolites displayed that the SCFAs are enriched in the majority of patients (at least 80%) (132). Similarly, a growing number of studies found that SCFAs directly exerted positive effects on DN, including attenuation of renal damage, prevention of insulin resistance, and renal function impairment (84, 90, 133).

The underlying molecular mechanism of the protective effect of SCFAs is worthy of further exploration and draws more attention. For instance, butyrate absorption by the intestinal epithelium is the major energy source for AMPK phosphorylation and promotion of glucagon-like peptide-1 (GLP1) release. Acetate and propionate exert essential functions through binding to G protein-coupled receptor 41 (GPR41) or G protein-coupled receptor 43 (GPR43) expressed on the intestinal epithelium. Activation of GPR41 promotes the secretion of peptide YY (PYY), which controls satiety and intestinal transport. In addition, GPR43 inhibits pro-inflammatory factor production and enhances the secretion of GLP1, which contributes to pancreatic β-cell proliferation, and thus, by lowering blood glucose levels, exerts a protective effect against DN (134). Decreased concentrations of SCFAs lead to decreased secretion of PYY and GLP1, thereby accelerating the development of DKD manifested by proteinuria, loss of renal structural integrity, and renal fibrosis (135). Furthermore, oxidative stress and NF-κB signaling were evidently activated in the pathophysiological process of DN, which could be significantly reversed by SCFAs. Moreover, the caspase1-GSDMD canonical pyroptosis pathway was also induced in the *in vitro* model treated with high glucose and presented a downregulated trend after SCFA intervention (136). In addition, several studies have shown that the activation of FFA2-mediated PI3K/Akt/mTOR signaling and the miR-7a-5p/P311/TGF-β1 pathway contributes to the positive regulation of SCFAs in DN (133, 137). In general, hyperglycemic stimulation triggers multiple factors to be disturbed, leading to intrarenal cellular abnormalities. Renal tubular injury is one of the important determinants of progressive renal failure in DN, and SCFAs significantly inhibited renal tubular cell apoptosis and oxidative stress induced by elevated glucose or H_2_O_2_ stimulation in *in vitro* experiments (138). Although considerable studies have demonstrated the protective effect of SCFAs on DN, there are still some controversies in this field, and their specific mechanisms in renal tubular cells, podocytes, and mesangial cells are not clear. For instance, it is suggested that increased levels of acetate related to disturbed gut microbiota enhance proteinuria, activate the renin-angiotensin-aldosterone system (RAAS), and aggravate renal intrinsic cell injury (139). The activation of RAAS is considered to be one of the important initial factors in the early development of DN. Acetate does not exist in germ-free mice. Under the stimulation of harmful factors, the intestinal flora will produce excessive short-chain fatty acids such as acetate, mediating the immune disorder and chronic inflammatory response of the host (140, 141). Acetate shows a dual role in DN. On the one hand, acetate can resist the damage of renal cells by external stimuli (such as anti-apoptosis and antioxidant stress) (138). On the other hand, SCFAs (such as acetate) can bind to receptors on renal arterioles and regulate renin secretion, participating in maintaining glomerular pressure. Due to the characteristics of early glomerular hypertension and glomerular ultrafiltration in DN, the disordered intestinal flora may produce excessive SCFAs, thus promoting the pathological changes of early DN (102). However, the role of SCFAs in DN still needs to be further investigated to provide more clinical and basic experimental evidence and to deeply explore its related mechanisms.

### 4.2 Obesity-related nephropathy

Obesity-related nephropathy (ORG) is a condition in which extreme obesity causes proteinuria, as first described by Weisinger et al. in 1974 (142). Obesity and CKD remain public health problems, and high BMI is an independent risk factor for the development of new-onset CKD. In obese patients, to meet the metabolic demands of the increased body weight, compensatory ultrafiltration occurs, increasing the intraglomerular pressure and damaging the kidneys through mechanisms such as lipotoxicity, chronic inflammation, and insulin resistance, increasing the risk of developing CKD (143). Recent evidence has highlighted other factors, including hemodynamic changes and gut microbiota dysbiosis, which exacerbate kidney dysfunction in obese patients, leading to histological changes known as ORG (144). Subsequently, an increasing number of clinical and experimental animal research studies focused on exploring the relationship between obesity and kidneys and showed that obesity substantially played a role in the structure and function of the kidney (145). Obese patients exhibited a faster reduction in glomerular filtration rate, making them more likely to develop ESRD or even death. Treating obesity proceeds to improve the aforementioned renal outcomes and significantly enhance the quality of life for patients (146). However, the underlying molecular mechanism of the ORG still remains unclear. It is commonly assumed to be linked to hyperglycemia and insulin resistance, the role of adipocytokines, irregular activation of RAAS, release of inflammatory factors, aberrant lipid metabolism, and renal structural damage caused by obesity itself (147). It was suggested that imbalanced gut microbiota and aberrant metabolites contribute to the progression of obesity, which is linked to the modulation of energy homeostasis, fat accumulation, and decreased lipoprotein lipase activity (148). High-fat diet (HFD) was utilized in feeding germ-free rodents to establish obesity-related changes in gut microbiota (149). Compared to the healthy group, microbial diversity was reduced in obese individuals (150, 151). A systematic review and meta-analysis showed that obese individuals had significantly higher SCFA levels and reduced richness of gut microbiota at the phylum level (152). This suggests that we cannot single-handedly use high or low levels of SCFAs to judge the status of obese patients, and to some extent, SCFAs may play an unfavorable role in obesity. In addition, the type of diet and the possibility of the genetic background of the experimental animals may also lead to different results in the data related to obesity-induced changes in the gut microbiota profile (153). For example, the investigation of children suggested that excessive SCFAs produced by a particular gut microbiota represent an additional energy source and may cause a disturbance of energy balance, contributing to obesity (154). Besides, a study of 441 community-dwelling adults reported that higher SCFA concentrations were associated with gut abnormal permeability, metabolic disturbance, and obesity (155).

SCFAs contributed to the inhibition of diet appetite, metabolic rate improvement, and loss of weight in mice and humans because of increasing release of leptin from adipose tissue (156). Endocrine hormones, such as GLP-1, PYY, and leptin, produced by the combination of SCFAs and GPRs, can increase satiety and improve obesity. Animal experiments found that inulin could stimulate the production of SCFAs in wild-type or FFAR2-/- mice, drive an increase in the number of cells that secrete PYY, increase the release of PYY, and then suppress appetite to control obesity (157). However, PYY can also slow down intestinal peristalsis, causing food to stay in the intestine for a longer time and increase energy absorption, thus leading to obesity. Increased GLP-1 hormone increases insulin sensitivity and inhibits fat accumulation in adipose tissue, thereby maintaining energy homeostasis in the body (158). SCFA production can promote leptin secretion from adipocytes by activating GPR43 receptors. In a mouse model of diet-induced obesity, the expression of GPR43 in adipose tissue is downregulated by SCFAs (159). In addition, GPR43-deficient mice prefer to gain weight regardless of diet, while overexpressing GPR43 in adipose tissue could reverse the phenomenon independent of HFD (160). However, in the fasting state, gastric tissue-derived leptin can promote appetite by attenuating the sensitivity of afferent nerves. It can be seen that leptin promotes satiety or appetite, depending on the state of food intake. Thus, it is obvious that obese patients need to increase the expression of GLP-1, PYY, leptin, and GPR43 for adaptive purposes, which may be responsible for the feedback elevation of SCFAs. Moreover, alterations in some of the key downstream components may also directly affect organismal performance through other pathways. For example, dietary SCFA supplementation prevented and reversed HFD-induced obesity in mice by downregulating PPARγ. Notably, the SCFA effect on lost weight was abrogated in mice with adipose-specific disruption of PPARγ, indicating that PPARγ acted as a critical mediator of the beneficial effects of SCFAs in MetS (160). Collectively, the factors contributing to obesity in the body are complex, involving genes, environment, and comorbidities. Generally, differences in diet, host genes, or the composition of the microbiota can also lead to certain differential manifestations of SCFAs in obese patients. There is still some controversy about whether SCFAs are beneficial or detrimental to obesity between humans and rodents; further studies should be conducted to validate the effect of SCFAs and the underlying mechanism.

### 4.3 Hypertension-related nephropathy

Hypertension-related nephropathy (HN) is a condition in which sustained hypertension causes damage to renal structure and function. High blood pressure increases the blood pressure in the blood vessels, resulting in the leakage of protein into the urine and causing damage to the renal filter system. Prolonged, poorly controlled hypertension further causes irreversible kidney damage. The initiation and progression of hypertension are attributed to dysregulation of the RAAS, ANS, and immune system (161, 162). Emerging evidence indicates that the gut microbiota and its metabolism play a role in hypertension development (163–165). The reduction of SCFAs-producing bacteria changed the gut environment, involving a decrease of the hypoxic gut profile and deterioration of the microbial balance, resulting in damage to epithelial barrier integrity, gut inflammation, dysregulation of blood pressure, and impairment of renal function. Consequently, impaired renal function leads to the accumulation of uremic toxins that reach the intestine and cause alterations in bacteria composition, which induces positive feedback that triggers the endotoxins to translocate into the bloodstream, enhances local kidney inflammation, and exacerbates kidney injury (166). In hemodialysis patients with renal disorders, sodium propionate supplementation resulted in a 10% drop in systolic blood pressure, while diastolic blood pressure remained constant (167). Dysbiosis of the intestinal flora has been reported in corresponding animal models of hypertension (168, 169) and in hypertensive patients (170, 171). For instance, spontaneously hypertensive rats (SHRs) displayed pathophysiological changes in the gut, including decreased numbers of goblet cells and villi length and increased fibrosis, compared to age-matched normotensive Wistar Kyoto (WKY) controls (172). Increasingly studies demonstrated the direct effect of gut dysbiosis on the initiation and progression of hypertension; specifically, fecal microbiota transplantation (FMT) experiments were conducted, including transferring dysbiotic fecal samples from patients with hypertension to germ-free mice (171) or feces from hypertensive stroke-prone SHRs to normotensive WKY rats, resulting in increased blood pressure in the recipients (173). The male SHR model showed that intestinal flora and its metabolites are closely associated with hypertension-related nephropathy, and the results indicate that reduced levels of SCFAs, inflammatory factor release, and blood pressure disturbances occur in the process (174). Mechanistically, a variety of SCFA receptors are seen to be expressed in human kidneys, and it was found that olfactory receptor 78 (Olfr78) was expressed mainly on afferent small arterioles (paraglomerular apparatus), which can elevate blood pressure by mediating renin secretion and subsequent vasoconstriction (165). SCFAs such as butyrate can target the GPR41 and olfactory receptor 78 (Olfr78) to prevent programmed hypertension by regulating cAMP (102, 175). Moreover, butyrate could attenuate DOCA/salt-induced hypertension and renal damage by inhibiting the MR/SGK1 pathway (176). Thus, it is clear that in HN, SCFAs can function through Olfr78, MR, etc., in addition to their usual function of binding to GPRs. However, it is obvious that other SCFA receptors, such as GPR41, are expressed in the renal microvascular system (especially the smooth muscle cells of small resistance vessels), but there is still some controversy about their role in regulating blood pressure (177–179). Currently, non-coding RNAs have become a hotspot in medical research; for example, microRNA (miRNA) is a kind of endogenous non-coding RNA with regulatory functions in eukaryotic organisms. miRNAs have been found to play a role in the regulation of blood pressure by binding to SCFAs receptors. For example, in hypertension-related nephropathy, miR-329 and miR-132 are upregulated, whereas miR-129 is downregulated. Furthermore, miR-329 and miR-132 can target GPR41 and GPR43, respectively, while miR-129 is expected to potentially target Olfr78 (180, 181). Despite the emerging evidence that SCFAs may play a role in the development of hypertension-related nephropathy, the underlying mechanisms of regulation of these receptors remain unclear and will require further studies to elucidate.

### 4.4 Hyperuricemia-related nephropathy

Uric acid (UA) is the end product of purine metabolism by xanthine oxidase, which belongs to anionic organic acid and is slightly soluble in water. Approximately 70% of UA in the normal human body is excreted through the kidney, and the remaining 30% is excreted through the bile duct and intestine (182). The increased generation or decreased excretion of UA results in the accumulation of UA in the body, and disturbances in UA in individuals will lead to hyperuricemia (HUA). HUA often contributes to renal dysfunction, for example, renal tubulointerstitial inflammation, kidney stones, renal fibrosis, and polycystic kidney disease (183). A study reported that the prevalence of nephropathy was 15.11% among 266 patients with HUA, while that was only 2.19% in the population with normouricemia (184). An investigation was conducted to explore the relationship between UA and subsequent decline in renal function. 13,338 participants with intact renal function in a community cohort were followed up and analyzed, and it was found that elevated serum UA level is an independent risk factor for kidney disease in the general population (185). Interestingly, it is reported that asymptomatic HUA could not affect CKD progression unless UA crystallizes and is deposited in kidney tissues. Following the development of UA crystal granulomas, renal interstitial inflammation, and fibrosis contribute to CKD progression, involving M1-like macrophage polarization (186). Currently, relevant studies have suggested a close relationship between the gut microbiota and HUA. A variety of obligate anaerobic and facultative anaerobic human and murine gut microbiota (mainly Firmicutes) have a higher ability to lower UA. This microbiota can drive the conversion of UA into lactic acid or anti-inflammatory SCFAs. Animal studies have shown that when there is a lack of hepatic uricase in the body, the gut microbiota structure changes significantly and the cecal and serum UA levels increase significantly (187). Moreover, HUA-related nephropathy is found to be usually accompanied by disordered intestinal flora, such as the overgrowth of opportunistic pathogens in HUA-related nephropathy, including *Escherichia-Shigella* and *Bacteroides*, and reduction of bacteria-producing SCFAs, such as *Lactobacillus* and *Ruminococcaceae* (188). The gut bacterial diversity and SCFAs in the HUA group reduced significantly, and the structure and function of the gut microbiota and the levels of SCFAs between patients with HUA and healthy controls have altered apparently (189). There are a large number of intestinal bacteria in the intestine that can consume UA under anaerobic conditions and convert it into xanthine or lactic acid and SCFAs, thereby changing the production and excretion of UA. The intestine has a variety of UA transporters, such as adenosine triphosphate-binding cassette Transporter G2 (ABCG2) and urate transporter soluble carrier protein 2 family member 9 (SLC2A9) (190). Then, what role do SCFAs play in HUA-associated nephropathy? Similarly, an animal research showed that the gut microbiota dysbiosis and decreased production of SCFAs in HUA-related nephropathy, and further experiments with FMT demonstrated that an increase in SCFAs could alleviate HUA-associated renal injury (66). Furthermore, an extra supplement of SCFAs could prevent cardiorenal lipotoxicity and glucometabolic dysregulation by suppressing UA accumulation (191). Mechanistically, several studies indicated that renal osteopontin, CD44, and TLR4/MyD88/NF-κB signaling were involved in the renal damage of HUA (192, 193). Overall, for the treatment and prevention of HUA-related nephropathy, previous studies inspire us not only to focus on the basic treatment of UA but also to pay attention to the reduction of SCFAs caused by diet and intestinal flora disorders of the patients. Moreover, the effort to find the therapeutic targets from multiple dimensions may provide new ideas for the prevention and treatment of HUA-related nephropathy.

## 5 Potential interventions

### 5.1 Diet

In recent years, studies have suggested that diet is closely related to the gut microbiota (194). Previous research analysis found that there are differences in the gut microbiota structure between healthy groups and CKD patients. CKD patients showed a decrease in the abundance of *Firmicutes* (128). Some studies have shown that supplementing dietary fiber can optimize the gut microbiota structure, promote the growth of glycolytic bacteria, increase the production of SCFAs, reduce gut microbiota-derived uremic toxins (such as p-cresol sulfate (p-CS), indoxyl sulfate (IS), and trimethylamine N-oxide), and have beneficial effects such as maintaining intestinal barrier integrity and mucus production to resist inflammation (195). Further animal studies have shown that a high-fiber diet can reduce kidney injury in animal models of MetS-related kidney disease (90, 196). However, there is currently a lack of relevant clinical studies to confirm the direct relationship between diet and the gut microbiota, and it is not clear how these diets affect the gut microbiota.

### 5.2 Probiotics, prebiotics, synbiotics, or postbiotics

Some research scholars have gradually paid attention to the prevention and treatment applications of gut microbiota and their metabolites in CKD (197). For example, well-known ones include probiotics (gut microbiota beneficial to the host), prebiotics (metabolites of gut microbiota beneficial to the host), synbiotics (gut microbiota and metabolites beneficial to the host), etc. Some studies have demonstrated through animal experiments that after the probiotic intervention, kidney inflammation and tubular cell apoptosis in mice with acute kidney injury were alleviated. Subsequently, a 1-year clinical trial was also conducted, and the results showed that probiotics slowed down the decline of kidney function in individuals with stages 3–5 CKD, both acute and chronic kidney injuries (106). A meta-analysis on the impact of probiotics on chronic kidney disease indicated that probiotics might also reduce the level of p-CS and increase the level of interleukin-6 (IL-6), thereby improving gastrointestinal symptoms (198). However, the impact of probiotics on the long-term prognosis of CKD patients remains to be studied. Additionally, a network meta-analysis of probiotics, prebiotics, and synbiotics in dialysis patients was conducted, and the results showed that prebiotics were the most effective in reducing tumor necrosis factor-α, urea, IS, and IL-6 (199). There are also newly emerging postbiotics (certain specific gut microbiota and their metabolites beneficial to the host), but the evidence regarding the application of postbiotics in kidney diseases is relatively lacking, and further research is needed (200).

### 5.3 FMT or smart bacteria

FMT refers to the transplantation of gut microbiota samples from healthy individuals into individuals with dysbacteriosis. In recent years, studies have found that FMT can alleviate the progression of kidney diseases (201). However, the vast majority of these studies were conducted in animal experiments, and the therapeutic efficacy has not been evaluated in clinical patients. There are relatively many applications of FMT in clinical MetS, such as diabetes, obesity, and gout (202). However, the research on FMT in MetS-related kidney diseases is still insufficient, and further studies are needed to confirm it. Additionally, some studies have found that intelligent bacteria can improve the gut microbiota structure of the body, reduce the level of uremic toxins in the body, and thus delay the progression of kidney diseases. For example, after oral administration of microcapsules containing *Escherichia coli* DH5 with urease-producing ability in uremic rats, the serum urea level decreased significantly (203). However, so far, no human studies have been conducted, and the issues of safety and biosafety have not been resolved (204).

### 5.4 SCFAs

SCFAs play important roles in influencing the body's energy homeostasis, immune function, and microbial signal transduction and have also become key risk factors affecting the development and prognosis of CKD (13). In recent years, studies have confirmed the potential therapeutic effects of SCFAs in patients with CKD. It has been found that during the process of CKD, the levels of fecal propionate and butyrate in patients gradually decrease. Through genome-wide expression assay analysis, it was found that propionate and butyrate jointly downregulated the expression of 103 genes related to the inflammatory process of tubular cells triggered by TNF-α and the activation of the immune system. Administration of propionate and butyrate either before or shortly after kidney injury in animal models prevented the occurrence and progression of kidney injury (110). Administration of butyrate intervention in a CKD mouse model has been shown to reduce kidney fibrosis (109). Interestingly, some researchers have proposed combining SCFAs with monotherapy, with SCFAs acting as drug carriers. SCFAs act on host gene expression by inhibiting HDAC inhibition (113). However, many studies have not focused on this area, and further research is still needed on the molecular mechanisms involved in the treatment of kidney diseases by SCFAs.

## 6 Conclusion

**Figure 3:**
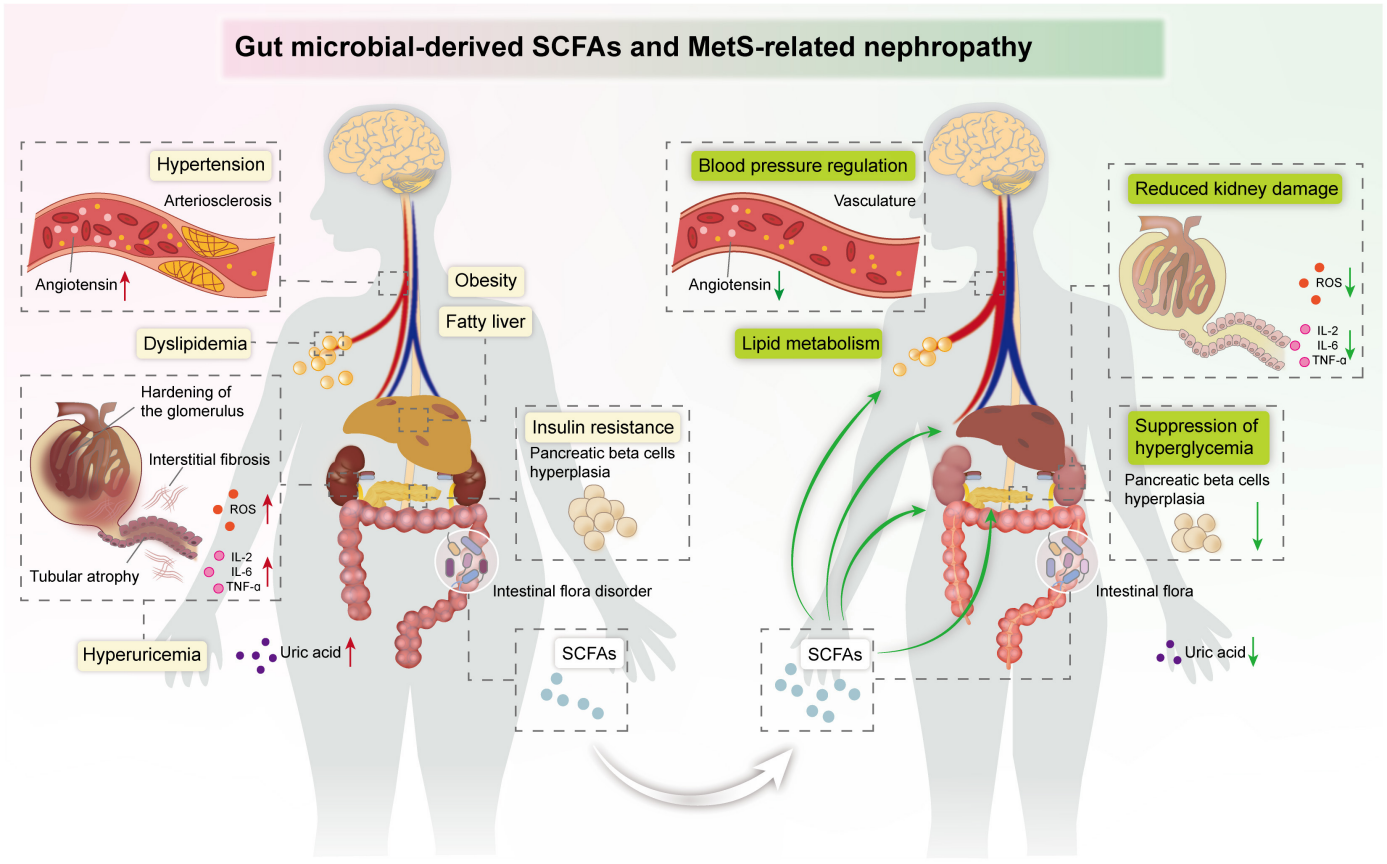
Summary image: SCFAs exert beneficial effects against kidney damage in the host, including regulation of blood pressure and lipid metabolism, suppression of hyperglycemia, and improvement of insulin resistance.

MetS is a group of clinical syndromes with chronic inflammation and metabolic disorders, which is an independent risk factor for CKD. Recently, the function of gut microbiota-derived SCFAs in kidney diseases has become an exciting area. Emerging evidence suggests that SCFAs have a wide range of roles in MetS-related nephropathy, including reprogramming of lipid, glucose, and UA metabolism, modulation of immune inflammation, and regulation of blood pressure. Existing clinical studies and animal experiments have indicated that SCFA treatment has potential therapeutic effects in MetS-related nephropathy, which provides prospective treatment of MetS-related nephropathy (Figure 3).

However, the specific role of SCFAs in MetS-related nephropathy has not yet been fully elucidated. The limitations of existing research are mainly reflected in the following aspects: First, most studies are based on animal models or clinical studies of small samples, lacking large samples and multi-center randomized controlled trials. Second, there are still some unknown issues regarding the specific mechanism of action of SCFAs, such as whether different types of SCFAs have different effects on the kidneys and how host genetics influence SCFA-mediated protection. In addition, the impact of diet-microbiota interactions (e.g., high-fat vs. high-fiber diets) on SCFA production, the interaction between SCFAs and other factors (such as diet, genetics, etc.), and its impact on the development of kidney diseases also need to be further explored. Therefore, future research directions should include more large-sample, multi-center, randomized controlled clinical trials, as well as in-depth research on the mechanism of action of SCFAs and their interaction with other factors. Furthermore, with the help of high-throughput sequencing technology and systems biology methods, the mechanism of action of different types of SCFAs in MetS-related nephropathy, as well as the impact of the overall structure and function of the gut microbiota on kidney disease, can be further investigated. The extensive efforts will allow us to gain a better understanding of the role of SCFAs in MetS-related nephropathy and give a more scientific basis for their clinical application.
